# Association between allostatic load and breast cancer risk: a cohort study

**DOI:** 10.1186/s13058-023-01754-w

**Published:** 2023-12-19

**Authors:** Yufan Guan, Jie Shen, Juan Lu, Bernard F. Fuemmeler, Lisa S. Shock, Hua Zhao

**Affiliations:** 1 Departments of Family Medicine and Population Health, School of Medicine, Virginia Commonwealth University, Richmond, VA 23284 USA; 2 Department of Public Health Sciences, School of Medicine, University of Virginia, Charlottesville, VA 22903 USA; 3 Departments of Microbiology and Immunology, School of Medicine, Virginia Commonwealth University, Richmond, VA 23284 USA

**Keywords:** Allostatic load, Breast cancer risk, Cohort study, Chronic stress

## Abstract

**Background:**

Allostatic load (AL) reflects the collective load of chronic stress during lifetime. Previous studies have shown that higher AL is associated with poor clinical outcomes among breast cancer patients. However, the relationship between AL and breast cancer risk is still unclear.

**Methods:**

To fill the gap, we analyzed the association between AL and the development of breast cancer in 181,455 women identified from the UK Biobank.

**Results:**

During the follow-up from 2006 to 2020, 5,701 women were diagnosed with incident breast cancer. Significantly higher AL was observed among incident breast cancer cases than all study participants (mean: 2.77 vs. 2.63, P < 0.01). Univariate Cox regression analysis indicated the risk of breast cancer was increased by 5% per one AL unit increase (hazard ratio (HR) = 1.05, 95% confidence interval (CI) 1.04, 1.07). In multivariate analyses, after adjusting demographics, family history of breast cancer, reproductive factors, socioeconomic status, lifestyle factors, and breast cancer polygenic risk score (PRS), the significant association remained (HR = 1.05, 95%CI 1.03, 1.07). The significant relationship was further confirmed in the categorical analysis. Compared with women in the low AL group (AL: 0 ~ 2), those in the high AL group (AL: 3 ~ 11) had a 1.17-fold increased risk of breast cancer (HR = 1.17, 95%CI 1.11, 1.24). Finally, in the stratified analysis, joint effects on the risk of breast cancer were observed between the AL and selected known breast cancer risk factors, including age, family history of breast cancer, PRS, income, physical activity, and alcohol consumption.

**Conclusion:**

In summary, those findings have demonstrated that higher AL was associated with an increased breast cancer risk in women. This association is likely independent of known breast cancer risk factors. Thus, the AL could be a valuable biomarker to help breast cancer risk prediction and stratification.

**Supplementary Information:**

The online version contains supplementary material available at 10.1186/s13058-023-01754-w.

## Background

In vitro cell line and animal-based studies have indicated that chronic stress may promote breast carcinogenesis [1–4]. However, epidemiological studies have shown conflicting results [5–9]. Thus, whether women who have experienced chronic stress have a higher risk of developing breast cancer remains to be determined. The discrepancy among epidemiological studies may be due to the difficulty of identifying and measuring chronic stressors and the weakness of the traditional chronic stress measurement method, such as (1) relying on questionnaires that are subjective to recall bias; (2) underestimating individuals' resilience; (3) lack of biomarkers representing chronic stress [10].

Research in the past decade has used the allostatic load (AL), a novel complex index involving cumulative physiological toll across multiple systems, to assess chronic stress [11, 12]. Results from those studies have suggested that the AL could be a key factor for understanding the physiological effects of long-term exposure to chronic stressors on chronic diseases, including cardiovascular, diabetes, cancer, etc. [13–15]. With breast cancer, several studies have explored how the AL may contribute to adverse breast cancer outcomes [16–21]. For example, high AL was associated with poor breast tumor differentiation and larger tumor sizes [19, 20], as well as poorer functional well-being and lower health-related quality of life among black women with breast cancer [21]. However, to date, there is no prospective study to determine the association between the AL and breast cancer risk. Our previous study found that higher AL was associated with an increased risk of overall cancer in women [22]. But, limited by the small sample size, we could not further assess the association for breast cancer risk.

In this study, we carried out the first study to investigate the association between AL and breast cancer risk, using the resource from the UK Biobank. We hypothesized that higher AL was associated with an increased breast cancer risk in women. In addition, we explored the joint effects between AL and known breast cancer risk factors.

## Methods

### Study population

The study population was identified from the UK Biobank, a prospective cohort study containing in-depth genetic and health information [23]. Detailed information on the UK biobank can be found at http://www.ukbiobank.ac.uk/. The study has followed over 500,000 volunteers in the UK, enrolled at ages 40–69 since 2006. For our study, we identified 181,455 female participants from the UK Biobank as the study cohort with a median follow-up time of 11.7 years (censored on 12/31/2020). We only included women without a history of cancer diagnosis at the time of enrollment. Women with a history of benign neoplasms, breast in situ carcinoma, and non-melanoma skin cancer were also excluded. In addition, we excluded women who had incomplete information on any of the eleven factors used to construct AL scores.

### Breast cancer cases ascertainment

In this study, we identified incident breast cancer cases using the ICD-10 code reported in the UK Biobank database (Additional file 1: Table S1). During the follow-up, incident breast cancer cases were defined as women newly diagnosed with malignant breast neoplasms. Only the first diagnosis was included for those with multiple breast cancer diagnoses. We excluded women diagnosed with breast cancer within one year after their initial enrollment. In total, 5,701 women with incident breast cancer were identified from the study population (N = 181,455).

### AL score construction

In this study, we used eleven factors to construct the AL score from measurements collected from the baseline. The detailed methods were described by Zhao and Chyu et al. [19, 22, 24]. Those factors comprised systolic blood pressure (SBP), diastolic blood pressure (DBP), C-reactive protein (CRP), high-lipoprotein cholesterol (HDL-C), low-density lipoprotein cholesterol (LDL-C), total cholesterol (TC), waist to hip ratio, triglycerides (TG), glycated hemoglobin (HbA1c), creatinine, and pulse rate (PR). The "abnormal cholesterol" was generated by combining LDL-C and TC. If the subject met either total cholesterol > 5.2 mmol/L or ≥ 5.2 mmol/L and LDL > 3.4 mmol/L, we considered the case as having abnormal cholesterol. Moreover, we also considered the history of medication for metabolic diseases and hypertension. The medication factor was interpreted as "Yes" by anyone with a metabolic disease or hypertension medication history. Therefore, the AL score included a total of eleven factors in the final, including three cardiovascular (SBP, DBP, PR), one inflammatory (CRP), six metabolic (HDL, waist to hip ratio, abnormal cholesterol, TG, HbA1c, creatinine), and one medication factor. This study categorized each selected factor as 1 or 0 based on the clinical risk threshold (Additional file 1: Table S2). The AL score was accumulated by all selected factors, ranging from 0 to 11. Higher scores represented higher levels of AL. The score was treated as a continuous variable and the categorical variables. For Categorical variable 1, we combined all categories with percentage less than 5% of all study participants into category 6. Thus, AL was categorized as 0, 1, 2, 3, 4, 5 and 6 to above, respectively. For Categorical variable 2, we used median level of AL in all study participants to divide the study population into two categories: 0–2 vs 3 to above.

### Covariates at baseline

At baseline, key characteristics included demographics (age and race), family history of breast cancer, reproductive factors (age of menarche, age of first live birth, history of contraceptive use, history of hormone-replacement therapy, and menopause status), socioeconomic status (education, employment status, income, and Townsend deprivation index), lifestyle factors (cigarette smoking, alcohol consumption, sleep quality, and physical activity) and breast cancer polygenic risk score (PRS). For any covariate with multiple measurements during the follow-up, we only included the measure recorded at the baseline (the time of recruitment into the UK Biobank). The Townsend deprivation index was calculated from previous national census output areas before participants joined the UK Biobank. Each participant was assigned a score corresponding to their postcode. Family history of breast cancer was accessed by whether one or two first-degree female relatives have had breast cancer. Education was classified as "high school or less" and "college/professional ". Income was classified as "less than £39,999)" and "over £39,999". Women's alcohol consumption was estimated by alcohol intake frequency, categorized as "special occasions or never," "moderate (less than twice per week)," and "heavy (more than three times per week)." Women's smoking status was derived using "current tobacco smoking" and "past tobacco smoking." Women's leisure physical activity was assessed based on MET scores and categorized into quintiles. Furthermore, women's sleep quality was assessed by whether they had sleeplessness or insomnia, categorized as "never/rarely, "sometimes," and "usually." The PRS for breast cancer was obtained from the UK biobank directly. Detailed information on how the PRS was generated has been described previous by Thompson et al. [25].

### Statistical analysis

First, we compared the difference in mean (for continuous variables) or distribution (for categorical variables) for each covariate between incident breast cancer cases and the overall study population. Next, we constructed the AL score using eleven factors and compared the distribution of the AL score between incident breast cancer cases and the general study population. AL was treated as continuous and categorical variables. The Student T-test or Chi-square test was used to detect the difference between continuous or categorical variables. Then, the Student T-tests were performed to assess the difference in AL in each category of a covariate between the overall study population and case groups. We also compared AL across the categories of each covariate within the general study population and case groups. The Student T-test or ANOVA was used to detect the difference between two or more two categories for each covariate. We applied univariate and multivariate Cox proportional hazard regression models to assess the association between the AL score and breast cancer risk. In this study, the enrollment date into the UK Biobank was the start time, and 12/31/2020 was used as the end of the study period. The event in this study was the first diagnosis of breast cancer. For breast cancer cases, the follow-up time was defined from the baseline to the date of breast cancer diagnosis. For those lost during the follow-up, the follow-up time was determined from the baseline to the date of the last follow-up. For those who developed other cancers (regardless of invasive or non-invasive), the follow-up time was defined from the baseline to the date of diagnosis of those cancers. Hazard ratios (HRs) and 95% confidence intervals (95% CIs) were estimated to assess the strength of the associations. The proportional hazards assumption was tested. If the assumption were violated, we would use the non-proportional hazards model instead. The Likelihood Ratio Test was used to assess the model fitting. In addition to the univariate analysis (Model 1), in the multivariate analysis, we built up a series of models with sequential adjustments of covariates, including demographic variables (age and race) in Model 2, demographic variables and family history of breast cancer in Model 3, demographic variables, family history of breast cancer, and reproductive factors (age when menarches, age of first live birth, contraceptive history, hormone-replacement therapy history, menopause status) in Model 4, demographic variables, family history of breast cancer, reproductive factors, and socioeconomic status (education, employment status, income, and deprivation) in Model 5, and demographic variables, family history of breast cancer, reproductive factors, socioeconomic status, lifestyle factors (smoking, alcohol consumption, sleeplessness, physical activity) and PRS in Model 6. Since the AL score was comprised of multiple components, we repeated the analysis to assess the association between each component and breast cancer risk. Finally, we explored the joint effect between AL and risk factors of breast cancer in this study population on the risk of breast cancer. Potential statistical interactions were noted. All statistical tests were two-sided, and p-values of less than 0.05 were considered statistically significant. The examinations were conducted using R, version 4.3.0.

## Results

A total of 181,455 women were included in the final analysis with a median of 11.7 years of follow-up. Within that, 5,701 incident breast cancer cases were observed. The median follow-up time for the cases was 6.21 years. Table 1 presents the distribution of the selected characteristics between cases and the overall study population. Compared to the general study population, breast cancer cases were more likely being older (56.85 vs. 56.11 years old, P < 0.01), White (95.72% vs. 94.41%, P < 0.01), having younger age of menarche (12.93 vs. 12.98, P = 0.03), ever used hormone-replace therapy (40.89% vs. 37.54%, P < 0.01), postmenopausal (61.95% vs. 58.96%, P < 0.01), having a family history of breast cancer (7.72% vs. 5.03%, P < 0.01), retired (36.43% vs. 33.90%, P < 0.01), ever smokers (41.40% vs. 39.65%, P < 0.01), less physically active (39.93% vs. 41.21%, < 0.01), heavy drinkers (40.48% vs. 36.74%, P < 0.01), and having higher PRS (0.38 vs. − 0.17, P < 0.01). No statistical significance difference was observed for age at first live birth, oral contraceptive pill use, education, income, Townsend deprivation score, and sleep quality.

**Table 1:** Selected characteristics in all cohort and breast cancer cases

	All cohort (N = 181,455)	Breast cancer case (N = 5,701)	P value
Age at recruitment, mean (SD)	56.11 (8.01)	56.85 (7.79)	< 0.01
Median follow-up for cases, Years		6.21	
Race, N (%)			< 0.01
White	171,379 (94.45%)	5,457 (95.72%)	
Black	2,791 (1.54%)	55 (0.96%)	
Asian	3,796 (2.09%)	106 (1.86%)	
Mixed or others	2,977 (1.64%)	66 (1.16%)	
Missing	512 (0.28%)	17 (0.30%)	
Age of menarches, mean (SD)	12.98 (1.62)	12.93 (1.62)	0.04
Age at first live birth, mean (SD)	25.42 (4.63)	25.49 (4.62)	0.07
Ever taken oral contraceptive pill, N (%)			0.93
Yes	147,276 (81.16%)	4,634 (81.28%)	
No	33,667 (18.55%)	1,056 (18.52%)	
Missing	512 (0.28%)	11 (0.19%)	
Ever used hormone-replacement therapy, N (%)			< 0.01
Yes	68,304 (37.64%)	2,331 (40.89%)	
No	112,578 (62.04%)	3,352 (58.80%)	
Missing	573 (0.32%)	18 (0.32%)	
Had menopause, N (%)			< 0.01
Yes	10,8513 (59.80%)	3,532 (61.95%)	
No	44,780 (24.68%)	1,285 (22.54%)	
Missing	28,162 (15.52%)	884 (15.51%)	
Family History of breast cancer, N (%)			< 0.01
Yes	10,3907 (57.26%)	3,141 (55.10%)	
No	9,274 (5.11%)	440 (7.72%)	
Missing	68,274 (37.63%)	2,120 (37.19%)	
Education, N (%)			0.14
High school or less	82,700 (45.58%)	2,584 (45.33%)	
College/professional	67,244 (37.06%)	2,174 (38.13%)	
Missing	31,511 (17.37%)	943 (16.54%)	
Employment, N (%)			< 0.01
Unemployment	16,706 (9.21%)	487 (8.54%)	
Employment	101,377 (55.87%)	3,094 (54.27%)	
Retired	61,651 (33.98%)	2,077 (36.43%)	
Missing	1,721 (0.95%)	43 (0.75%)	
Income, N (%)			0.51
< £30,999	75,416 (41.56%)	2,506 (43.95%)	
≥ £30,999	74,697 (41.17%)	2,262 (39.68%)	
Missing	31,341 (17.27%)	933 (16.37%)	
Townsend deprivation score (Z-score, mean/SD)	− 1.39 (3.00)	− 1.43 (2.93)	0.16
Cigarette smoking, N (%)			0.03
Never	108,780 (59.95%)	3,325 (58.32%)	
Ever	72,038 (39.70%)	2,360 (41.40%)	
Missing	634 (0.35%)	16 (0.28%)	
Total physical activity MET-hours/week ((Z-score, mean/SD))	42.02 (41.21)	39.61 (39.93)	< 0.01
Alcohol consumption, N (%)			< 0.01
Special occasions or never	43,976 (24.24%)	1,288 (22.59%)	
Moderate	70,460 (38.83%)	2,103 (36.89%)	
Heavy	66,878 (36.86%)	2,308 (40.48%)	
Missing	141 (0.08%)	2 (0.04%)	
Sleep quality, N (%)			0.48
Never/rarely sleepless	34,586 (19.06%)	1,071 (18.79%)	
Sometimes sleepless	89,381 (49.26%)	2,792 (48.97%)	
Usual sleepless	57,385 (31.62%)	1,837 (32.22%)	
Missing	103 (0.06%)	1 (0.02%)	
Standard PRS for breast cancer (Z-score, mean/SD)	− 0.17 (1.00)	0.38 (1.00)	< 0.01

The distribution of AL scores in the overall study population and breast cancer cases is presented in Table 2. The range of AL was from 0 to 11. On one side, no women had all 11 risk factors (AL = 11). On the other hand, the proportion of women with AL equal to 1 was the largest, accounting for approximately 24% of the study population. Overall, the distribution of AL presented a decreasing trend from 1 to 11, and only about 7% of women have an AL equally or greater than 6. A significant difference in the distribution of AL scores was observed between the overall study population and breast cancer cases (P < 0.01). Compared to the general study population, breast cancer cases were less likely to have AL scores of 0 and 1 and more likely to have AL scores of 3 and above. As a continuous variable, the mean AL score of the breast cancer cases was 2.77, statistically significantly higher than the 2.63 of the overall study population (p < 0.01). Then, we combined women with AL scores of 6 and above into one category and generated a new variable, AL Category 1, which included seven categories. A significant difference in AL Category 1 was observed between the study population and breast cancer cases (P < 0.01). Additionally, to further simplify the data, we generated a dichotomized variable, AL Category 2, which includes two groups: a low AL group with an AL from 0 to 2 and a high AL group with an AL from 3 to 11. A significant difference in AL Category 2 was observed between the study population and breast cancer cases (P < 0.01). Breast cancer cases were more likely in the high AL group than their counterparts (51.55% vs. 47.68%, P < 0.01).

**Table 2:** Distribution of AL scores and AL score category in all cohort and breast cancer cases

	All cohort (N = 181,455)	Case (N = 5,701)	P value
AL score			< 0.01
0	14,046 (7.74%)	376 (6.60%)	
1	42,882 (23.63%)	1,243 (21.80%)	
2	38,012 (20.95%)	1,143 (20.05%)	
3	33,111 (18.28%)	1,091 (19.14%)	
4	24,868 (13.70%)	871 (15.28%)	
5	16,214 (8.94%)	541 (9.49%)	
6	8,313 (4.58%)	278 (4.88%)	
7	3,139 (1.73%)	129 (2.26%)	
8	741 (0.41%)	24 (0.42%)	
9	121 (0.07%)	4 (0.07%)	
10	8 (< 0.01%)	1 (0.02%)	
11	0	0	
AL, continuous (mean/SD)	2.63 (1.74)	2.77 (1.76)	< 0.01
AL category 1			< 0.01
0	14,046 (7.74%)	376 (6.60%)	
1	42,882 (23.63%)	1,243 (21.80%)	
2	38,012 (20.95%)	1,143 (20.05%)	
3	33,111 (18.28%)	1,091 (19.14%)	
4	24,868 (13.70%)	871 (15.28%)	
5	16,214 (8.94%)	541 (9.49%)	
6 and over	12,322 (6.79%)	436 (7.65%)	
AL category 2			< 0.01
Low (0 ~ 2)	94,940 (52.32%)	2,762 (48.45%)	
High (3 ~ 11)	86,515 (47.68%)	2,939 (51.55%)	

Then, we compared the AL score between the overall study population and breast cancer cases by selected characteristics (Table 3). In general, compared to the overall study population, the mean AL for each selected characteristics was statistically significantly higher in the cases (P < 0.05), except for women who were younger than 57 years old, Black, Asian, mixed, or others, pre-menopausal and had less than 20% total physical activity MET (hours/week). Then, we compared the AL score by selected characteristics within the overall study population and cases separately (Table 3). Among the general study population, compared to their counterparts, women who were older than 57 years old, postmenopausal, retired, and had first live birth before age 30, age of menarche younger than ten years old, never taken oral contraceptive pill, ever used hormone-replacement therapy, no family history of cancer, high school or less education, income less than £30,999, less physically active, drunk alcohol rarely, and frequent sleeplessness had statistically significant higher AL score (P < 0.05). A similar trend was also observed among cases. Additionally, in breast cancer cases, the mean AL score differed among racial groups (P < 0.01). Asian and Black had higher AL scores than White breast cancer cases.

**Table 3:** Comparison of AL score by selected characteristics between all cohort and breast cancer cases

	AL, mean, (SD)		
	All cohort (N = 181,455)	Case (N = 5,701)	P value
Age at recruitment, median			
< 57 years old	2.16 (1.70)	2.20 (1.70)	0.23
≥ 57 years old	3.07 (1.67)	3.19 (1.68)	< 0.01
P value	< 0.01	< 0.01	
Race			
White	2.62 (1.73)	2.77 (1.76)	< 0.01
Black	2.87 (1.83)	2.87 (1.75)	0.96
Asian	3.05 (1.87)	3.18 (1.71)	0.46
Mixed or others	2.58 (1.73)	2.56 (1.73)	0.92
P value	0.06	< 0.01	
Age at first live birth			
< 30 years old	2.82 (1.74)	2.94 (1.74)	< 0.01
≥ 30 years old	2.16 (1.64)	2.39 (1.73)	< 0.01
P value	< 0.01	< 0.01	
Age at menarche			
< 10 years old	2.82 (1.74)	3.03 (1.85)	0.02
≥ 10 years old	2.16 (1.64)	2.61 (1.73)	< 0.01
P value	< 0.01	< 0.01	
Ever taken oral contraceptive pill			
Yes	2.55 (1.73)	2.69 (1.74)	< 0.01
No	2.97 (1.75)	3.11 (1.78)	0.01
P value	< 0.01	< 0.01	
Ever used hormone-replacement therapy			
Yes	2.97 (1.68)	3.09 (1.66)	< 0.01
No	2.43 (1.74)	2.55 (1.79)	< 0.01
P value	< 0.01	< 0.01	
Had menopause			
Yes	2.87 (1.68)	3.02 (1.69)	< 0.01
No	1.85 (1.61)	1.83 (1.58)	0.64
P value	< 0.01	< 0.01	
Family history of breast cancer			
Yes	2.72 (1.74)	2.84 (1.74)	< 0.01
No	2.50 (1.69)	2.72 (1.76)	0.01
P value	< 0.01	0.17	
Education			
High school or less	2.63 (1.73)	2.78 (1.74)	< 0.01
College/professional	2.32 (1.66)	2.49 (1.73)	< 0.01
P value	< 0.01	< 0.01	
Employment			
Unemployment	2.71 (1.85)	2.91 (1.94)	0.03
Employment	2.30 (1.68)	2.43 (1.71)	< 0.01
Retired	3.16 (1.66)	3.25 (1.66)	0.01
P value	< 0.01	< 0.01	
Income			
< £30,999	2.93 (1.75)	3.08 (1.77)	< 0.01
≥ £30,999	2.21 (1.77)	2.38 (1.67)	< 0.01
P value	< 0.01	< 0.01	
Cigarette smoking			
Never	2.56 (1.73)	2.71 (1.75)	< 0.01
Ever	2.77 (1.74)	2.91 (1.80)	< 0.01
P value	< 0.01	< 0.01	
Total physical activity MET-hours/week			
< 20%	2.87 (1.81)	2.93 (1.84)	0.30
20 ~ 40%	2.58 (1.74)	2.79 (1.74)	< 0.01
40 ~ 60%	2.47 (1.69)	2.56 (1.69)	0.14
60 ~ 80%	2.41 (1.67)	2.58 (1.67)	< 0.01
≥ 80%	2.44 (1.65)	2.57 (1.71)	0.04
P value	< 0.01	< 0.01	
Alcohol consumption			
Special occasions or never	3.06 (1.84)	3.26 (1.88)	< 0.01
Moderate	2.60 (1.74)	2.73 (1.75)	< 0.01
Heavy	2.38 (1.62)	2.53 (1.64)	< 0.01
P value	< 0.01	< 0.01	
Sleep quality			
Never/rarely sleepless	2.34 (1.71)	2.45 (1.72)	0.04
Sometimes sleepless	2.62 (1.72)	2.78 (1.75)	< 0.01
Usually sleepless	2.84 (1.76)	2.95 (1.76)	< 0.01
P value	< 0.01	< 0.01	

**Fig. 1:**
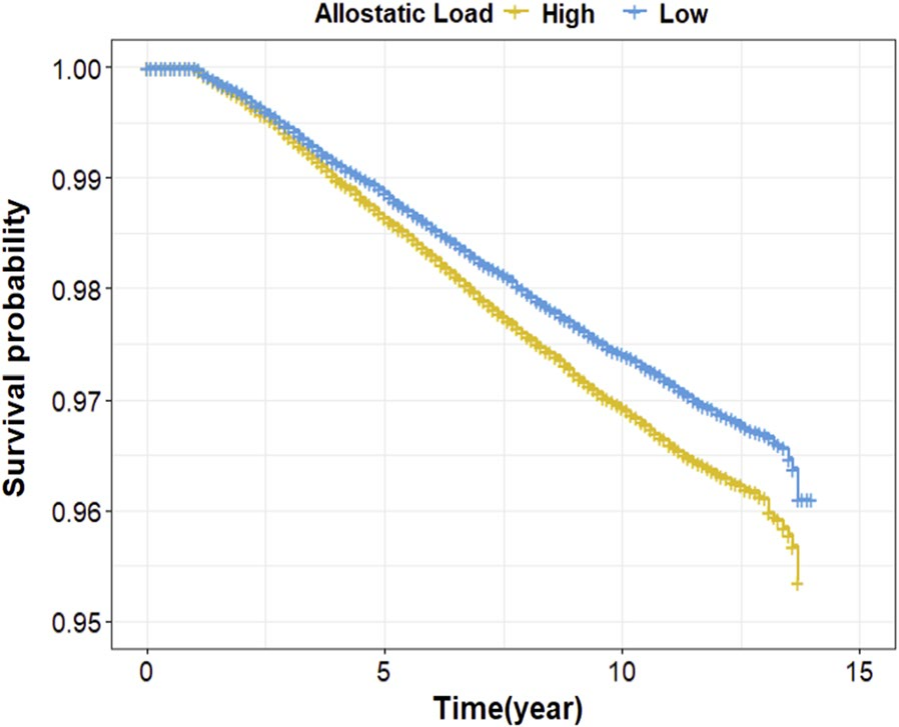
Kaplan–Meier survival estimates for the association between the AL score and breast cancer risk

Next, we investigated the association between AL and breast cancer risk (Table 4). Firstly, we treated the AL score as a continuous variable. Univariate Cox proportional hazard regression (Model 1) analysis indicated the risk of breast cancer was increased by 5% per one AL unit increase (HR = 1.05, 95%CI 1.04, 1.07). Figure 1 displays the Kaplan–Meier survival curves for the association between the AL score and breast cancer risk. Compared to those with low AL scores (0–2), those with high AL scores (> 2) had a statistically significant higher likelihood of developing breast cancer (p < 0.01). In further analyses, we built a series of models (from Models 2–6) with sequential adjustments of covariates. The Schoenfeld residuals testing suggested no statistically significant violation of each model's Cox proportional hazards regression model assumption. The association between AL score and breast cancer risk remained statistically significant in all five models (Model 2: HR = 1.04, 95%CI 1.02, 1.05; Model 3: HR = 1.04, 95%CI 1.02, 1.05; Model 4: HR = 1.04, 95%CI 1.02, 1.06; Model 5: HR = 1.05, 95%CI 1.03, 1.06; and Model 6: HR = 1.05, 95%CI 1.03, 1.07). We also performed similar analyses for AL Categories 1 and 2 (Table 4). For AL Category 1, statistically significant associations were observed for AL scores of Categories 3, 4, 5, and 6 consistently across six models, with an HR ranging from 1.24 (HR = 1.24, 95%CI 1.06, 1.41) for model 2–1.37 (HR = 1.37, 95%CI 1.19, 1.58) for model 1. In further trend tests, a statistically significant increasing trend of breast cancer risk was observed in each model (p for trend < 0.01, respectively). For AL Category 2, compared with the low AL group, the high AL group had a statistically significant increased risk of breast cancer in each model, with an HR ranging from 1.13 (HR = 1.13, 95%CI 1.07, 1.19) for model 2–1.19 (HR = 1.19, 95%CI 1.13, 1.25) for model 1.

**Table 4:** Associations between AL scores and AL categories with breast cancer risk

	Mode 1	Mode 2	Mode 3	Mode 4	Mode 5	Mode 6
AL, continuous, Per one unit	1.05 (1.04, 1.07)	1.04 (1.02, 1.05)	1.04 (1.02, 1.05)	1.04 (1.02, 1.06)	1.05 (1.03, 1.06)	1.05 (1.03, 1.07)
AL category 1						
0	Reference	Reference	Reference	Reference	Reference	Reference
1	1.10 (0.98, 1.23)	1.04 (0.92, 1.17)	1.04 (0.92, 1.17)	1.06 (0.94, 1.19)	1.06 (0.94, 1.19)	1.06 (0.94, 1.19)
2	1.15 (1.02, 1.29)	1.05 (0.93, 1.18)	1.06 (0.94, 1.19)	1.07 (0.95, 1.21)	1.09 (0.96, 1.23)	1.09 (0.96, 1.23)
3	1.27 (1.13, 1.43)	1.14 (1.02, 1.29)	1.15 (1.02, 1.29)	1.17 (1.04, 1.33)	1.19 (1.05, 1.35)	1.20 (1.06, 1.35)
4	1.36 (1.20, 1.53)	1.21 (1.07, 1.37)	1.22 (1.08, 1.38)	1.26 (1.11, 1.43)	1.28 (1.13, 1.46)	1.29 (1.14, 1.47)
5	1.29 (1.13, 1.47)	1.22 (1.01, 1.32)	1.16 (1.02, 1.33)	1.20 (1.05, 1.38)	1.24 (1.08, 1.42)	1.26 (1.09, 1.44)
6 and over	1.37 (1.19, 1.58)	1.24 (1.06, 1.41)	1.24 (1.08, 1.43)	1.28 (1.10, 1.47)	1.31 (1.14, 1.52)	1.34 (1.15, 1.55)
P for trend	< 0.01	< 0.01	< 0.01	< 0.01	< 0.01	< 0.01
AL category 2						
Low	Reference	Reference	Reference	Reference	Reference	Reference
High	1.19 (1.13, 1.25)	1.13 (1.07, 1.19)	1.14 (1.08, 1.20)	1.15 (1.09, 1.21)	1.17 (1.10, 1.23)	1.17 (1.11, 1.24)

Model 1: univariate

Model 2: adjusted by demographic variables

Model 3: adjusted by demographic variables and family history

Model 4: adjusted by demographic variables, family history, and reproductive factors

Model 5: adjusted by demographic variables, family history, reproductive factors, and SES

Model 6: adjusted by demographic variables, family history, reproductive factors, lifestyle factors, SES, and PRS

We also explored the association between each AL score’s component and breast cancer risk (Additional file 1: Table S4). After adjusting all covariates, we found higher levels of waist-to-hip ratio, SBP, DBP, and CRP, lower levels of HDL, and having a history of metabolic disease or hypertension medication were associated with an increased risk of breast cancer, respectively (waist-to-hip ratio: HR = 1.13, 95%CI 1.07,1.19; SBP: HR = 1.08, 95%CI 1.02,1.15; DBP: HR = 1.09, 95%CI 1.02,1.17; CRP: HR = 1.13, 95%CI 1.06,1.20; HDL: HR = 1.13, 95%CI 1.06,1.21; and A history of metabolic disease or hypertension medication: HR:1.07, 95%CI 1.01,1.14).

Finally, we explored the joint effect between AL and selected breast cancer risk factors associated with breast cancer risk in this study population, including age of recruitment, family history of breast cancer, PRS, income, total physical activity MET, and alcohol consumption (Table 5). Demographic variables, family history, reproductive, healthy behaviors, SES, and PRS, were adjusted in the model as appropriate. As expected, an addictive or multiplicative joint effect was observed for each selected risk factor except age of recruitment. Interestingly, a more than addictive or multiplicative joint effect was observed between age of recruitment and AL score (P < 0.01). Compared to women with low AL and younger age (< 57 years old), the risk of breast cancer was 1.08 (HR = 1.08, 95%CI 0.99, 1.18) for those with high AL and younger age, 1.24 (HR = 1.24, 95%CI 1.13, 1.38) for those with low AL and older age (≥ 57 years old), and 1.54 (HR = 1.54, 95%CI 1.40, 1.70) for those with high AL and older age.

**Table 5:** Joint effects of AL and breast cancer risk factors

	AL low	AL high
Age group *		
< 57 years old	Reference	1.08 (0.99, 1.18)
≥ 57 years old	1.24 (1.13, 1.38)	1.54 (1.40, 1.70)^1^
Family history of breast cancer		
No	Reference	1.15 (1.07, 1.24)
Yes	1.35 (1.17, 1.56)	1.81 (1.57, 2.09)
PRS		
< 75%	Reference	1.14 (1.06, 1.23)
≥ 75%	2.35 (2.18, 2.54)	2.87 (2.66, 3.10)
Income		
< £30,999	Reference	1.15 (1.06, 1.26)
≥ £30,999	1.06 (0.97, 1.16)	1.26 (1.15, 1.38)
Total physical activity MET-hours/week		
≥ 60%	Reference	1.19 (1.08, 1.32)
< 60%	1.20 (1.10, 1.31)	1.36 (1.25, 1.48)
Alcohol consumption		
Special occasions or never or moderate	Reference	1.17 (1.08, 1.25)
Heavy	1.13 (1.05, 1.22)	1.33 (1.23, 1.44)

*P for interaction < 0.01

## Discussion

Previous studies in breast cancer have shown the association between increased AL and poor tumor differentiation, larger breast tumor sizes, and worse prognosis [16–21]. However, the association between the AL and breast cancer risk is unknown. In this study, using valuable resources from the UK Biobank, we performed the first analysis to assess the relationship between AL and subsequent breast cancer risk. We found that higher AL was associated with an increased risk of breast cancer. With one AL unit increase, a 5% increase in breast cancer risk was observed (HR = 1.05, 95%CI 1.04, 1.07). More importantly, the risk association between AL and breast cancer seems independent of demographics, family history of breast cancer, reproductive factors, socioeconomic status, lifestyle factors, and PRS.

Several studies have assessed the relationship between stress and breast cancer risk, and the results are mixed [26–29]. On one side, in a cohort study of 10,808 Finnish women with over 15 years of follow-up, stressful life events were associated with an increased risk of breast cancer [26]. In another cohort of 1,462 Swedish women with 24 years of follow-up, higher levels of daily stress are associated with a twofold increase in breast cancer risk [27]. On the other side, a prospective cohort study of 106,000 women for a follow-up period of 6.1 years in the United Kingdom demonstrated a null association between breast cancer risk and perceived stress levels [28]. Furthermore, another European prospective study examining 11,467 women over ten years found no evidence that social stress was associated with breast cancer incidence [29]. In contrast to those studies mentioned above, which used questionnaire-based instruments to capture individual stressors, ranging from daily stressful life events to social stress, we have used AL in this study. Because AL not only reflects the cumulative wear and tear from chronic stress but also accounts for individual difference in resilience and biology, AL is thought suitable to be used as a biomarker to assess the relationships between chronic stress and chronic diseases. However, the association between AL and cancer risks has rarely been studied. The only exception is our previous report in the SWAN study, showing that a higher AL score is associated with increased overall cancer [22]. Unfortunately, due to small sample size, the association between AL and breast cancer risk could not been evaluated in the SWAN study. The current study in the UK biobank helped fill the gap.

The significant association between AL score and breast cancer risk is biologically relevant. Chronic stress continuously activates the sympathetic nervous system, significantly increasing stress hormone expression levels (e.g., glucocorticoids, epinephrine, norepinephrine) [30, 31]. Overexpression of stress hormones results in dysregulation of tumor-suppressor genes, such as *BRCA1* and *p53*, leading to increased DNA damage and tumorigenesis [32–34]. Moreover, stress hormones also, through complex mechanisms, cause disorders of the immune, cardiovascular, metabolic, and neuroendocrine systems and promote tumor cell proliferation, invasion, and metastasis [2, 35–37]. For example, glucocorticoids prevent immature dendritic cells from fully maturing, while fully maturing dendritic cells are essential in initiating cancer adaptive immunity [37]. Long-term glucocorticoid exposure can progressively cause visceral fat accumulation and insulin resistance [38]. Moreover, through adrenergic receptors, epinephrine and norepinephrine could promote breast cancer cell proliferation, migration, and invasion [39].

In our study, we found that levels of AL are significantly affected by demographics (age and race), family history of breast cancer, reproductive factors (age at first live birth, oral contraceptive pill, hormone replacement therapy, and menopausal status), socioeconomic status (education, employment, and income), and lifestyle factors (cigarette smoking, alcohol consumption, physical activity, and sleep quality) in either overall study population, breast cancer cases, or both. Interestingly, many of those factors are known breast cancer risk factors. As shown in Additional file 1: Table S3, older women, white, had a family history of breast cancer, and ever used hormone replacement therapy were more likely to develop breast cancer. We also found that women who were ever smokers, less physically active, and heavy drinkers had an increased risk of breast cancer. When put together, it might lead us to assume that the association between AL and breast cancer risk can be modified by those factors mentioned above. However, what we found from this study is the opposite. In the Cox regression analysis, the association between AL and breast cancer risk is consistent from univariate (Model 1) to multivariate (Models 2–6) analyses with an HR of 1.05 per one AL unit increase, indicating the effect of AL on breast cancer risk is independent of demographics, family history of cancer, reproductive factors, socioeconomic status, and lifestyle factors. A similar relationship was also observed in our previous analysis in the SWAN study that the significant association between AL and overall cancer risk was not affected by demographics, healthy behaviors, and SES factors [22]. Though the underlying mechanism is unclear, our data from this study have suggested that the biological pathway linking chronic stress and breast cancer development is likely independent of other known breast cancer risk factors. To further support the notion, we found the risk association was consistent in individual categories for nearly all covariates (Table 3). In addition, we observed joint effects between AL score and several breast cancer risk factors, including age, family history of breast cancer, PRS, income, physical activity, and alcohol consumption (Table 5).

For each covariate, a significant difference in AL score was observed across categories in the overall study population and/or breast cancer cases. The difference in age group, education, income, cigarette smoking, physical activity, alcohol consumption, and sleep quality is consistent with previous reports from our own and others [19, 22, 24]. Previous literature reports have shown that women who had an earlier age of first live birth had higher levels of AL [40–42]. The results from this study support the notion. We found that women with younger age of first live birth (≤ 30 years old) had elevated AL scores than those with older age of first live birth (> 30 years old) in both the overall study population and the breast cancer cases (P < 0.01, respectively). This may be due to the cumulative impact of early childbearing and a higher risk of pregnancy complications among young mothers [41]. We also found that women with younger age of menarche (< 10 years old) had higher levels of AL. This is consistent with a previous report from Allsworth et al. [42]. In addition, we found women who had never taken oral contraceptive pills and had ever used hormone-replacement therapy had higher AL scores than their counterparts. However, the associations were not significant anymore after adjusting other covariates.

In Additional file 1 Table S4, we have observed significant associations between several components of the AL score and breast cancer risk. Given the nature of those factors (e.g., waist-to-hip ratio, SBP, DBP, CRP, HDL, and a history of metabolic disease or hypertension medication), it is assumed that suboptimal cardiovascular, metabolic, and immune systems may increase the risk of breast cancer. Interestingly, as behavioral and pharmacologic interventions are available to improve cardiovascular, metabolic, and immune functions, those interventions may be valuable for breast cancer risk reduction, particularly among those with higher AL scores.

One surprising finding is that women with a family history of breast cancer had lower levels of AL than their counterparts in the overall study population (P < 0.01). The significant difference remained even after the adjustment of age and other covariates. To date, no study has assessed the relationship between the family history of breast cancer and AL in the population. However, several studies have reported that women with a family history of cancer or with *BRCA1/2*-positive had higher levels of psychological stress than their counterparts [43–45]. One study has also indicated that the coping style further modified the association [45]. One discrepancy between our and their studies is the measure of stress. Another discrepancy is the study population. Previous studies have small sample sizes (60–80 women), and the study subjects were recruited from special settings (e.g., cancer clinics and online *BRCA* supporting groups). So, the results from those studies may differ from those from population-based large cohort studies, such as UK Biobank. More research is needed in the future to clarify the association further.

There were two major limitations in this study. First, given about 95% of the study population is White, this study lacks minorities in the study population. This may limit the generalizability of the findings in more diverse populations. Since minorities are known to have higher levels of chronic stress than their white counterparts, the research on minorities is significant. Second, the UK Biobank lacks information on breast tumor subtypes at this moment. Breast tumors are heterogeneous. Different tumor subtypes may have different etiologies. So, the risk association between AL and breast cancer risk may only exist in particular breast cancer subtypes.

## Conclusions

In summary, we carried out one of the first studies to assess the role of AL on the risk of breast cancer in a large cohort. We found that higher AL was associated with an elevated risk of breast cancer in women. This association tends to be independent of demographics, reproductive factors, family history of cancer, SES, lifestyles, and PRS. Thus, AL, as a biomarker of chronic stress, could be useful in breast cancer risk stratification and prediction. Other cohort studies must further confirm the results, particularly those with a large number of minorities.

### Supplementary Information

**Additional file 1: Table S1.** Codes used to identify breast cancer cases (Study censoring date: 12/31/2020). **Table S2.** Distribution and high-risk cutoff points for individual biomarkers of AL scores. **Table S3.** Association of breast cancer risk with allostatic load (N=181,455). **Table S4.** Association between individual biomarkers of AL scores and breast cancer risk.

## Data Availability

All data generated or analyzed during this study are included in this published article and its supplementary information files.
