# Supplementary material for: Association between allostatic load and breast cancer risk: a cohort study
Source: Breast Cancer Res. 2023 Dec 19;25:155. doi: 10.1186/s13058-023-01754-w (PMC10729373; doi:10.1186/s13058-023-01754-w)
Supplement: Supplementary file 1 — Additional file 1: Table S1. Codes used to identify breast cancer cases (Study censoring date: 12/31/2020). Table S2. Distribution and high-risk cutoff points for individual biomarkers of AL scores. Table S3. Association of breast cancer risk with allostatic load (N=181,455). Table S4. Association between individual biomarkers of AL scores and breast cancer risk. [file 13058_2023_1754_MOESM1_ESM.docx]

**Supplementary Tables:**

Table1 : Codes used to identify breast cancer cases (Study censoring date:12/31/2020)

| **Categories** | **Frequency (%)** | **ICD10 codes** |
| --- | --- | --- |
| **Breast cancer cases** | | |
| - Prevalent: | 7998 (2.93%) | Codes start with C50 and its subclasses , C501, C502, C503, C504, C505, C506, C507, C508, and C509 |
| - Incident: | 8416(3.08%) [7594(Incident >1 year);  822(Incident <1 year))] |  |
| **Other cancers(except for non-melanoma skin cancer )** | | |
| - Prevalent: | 5867(2.15%) | Codes start with C except codes for BC and non-melanoma skin cancer |
| - Incident: | 13674(5.00%) |  |
| **In situ carcinoma** | | |
| - Prevalent: | 5701(2.09%) | Codes start with D0-D09 |
| - Incident: | 3473(1.27%) |  |
| **Neoplasm of unknown nature or behavior** | | |
| - Prevalent: | 569(0.21%) | Codes start with D37- D48 |
| - Incident: | 848(0.31%) |  |
| **Benign neoplasms** | | |
| - Prevalent: | 421(0.15%) | Codes start with D10-D36 |
| - Incident: | 432(0.16%) |  |
| **non-melanoma skin cancer** | | |
| - Prevalent: | 4343(1.59%) | Codes start with C44 |
| - Incident: | 10307(3.77%) |  |
| **Unknow prevalent cancers** | 5059(1.85%) | Subject only with cancer diagnosed time, no Codes |
| **Non-case controls** | 206193 (79.45%) | Remaining codes or subjects with no code assigned |
| **Total** | 273298 (100%) | |

- Subject with any cancer developed before enrolment was excluded.
- Subject with any cancer other than breast cancer during follow-up were censored at the date of diagnosis.

Table2 : Distribution and high-risk cutoff points for individual biomarkers of AL scores (N=242521)

|  | Cutoff Value | Mean(SD) | N (%) at Risk | N (%)missing |
| --- | --- | --- | --- | --- |
| Waist to hip ratio | >=0.85 for female | 0.82(0.07) | 74913(30.89%) | 990(0.41%) |
| Pulse rate | >100 | 70.12(10.57) | 2110(0.87%) | 14674(6.05%) |
| SBP (mm Hg) | >=140 | 135.05(19.21) | 83726(34.52%) | 14676(6.05%) |
| DBP (mm Hg) | >=90 | 80.66(10.01) | 40918(16.87%) | 14674(6.05%) |
| HDL (mmol/L) | >1.3 for female | 1.59(0.38) | 46784(19.29%) | 36803(15.18%) |
| LDL ( mmol/L) | >3.4 | 3.62(0.87) | 129797(53.52%) | 16593(6.84%) |
| Total Cholesterol(mmol/L) | >5.2 | 5.86(1.12) | 160869(66.33%) | 16227(6.69%) |
| Triglycerides(mmol/L) | >=1.7 | 1.54(0.85) | 71341(29.42%) | 16348(6.74%) |
| C-reactive protein( mg/L) | >3 | 2.67(4.28) | 55183(22.75%) | 16608(6.85%) |
| Creatinine(umol/L) | >97.2 for female | 64.28(13.26) | 2255(0.93%) | 16325(6.73%) |
| Glycated hemoglobin (HbA1c)mmol/mol | >48 | 35.75(5.97) | 5861(2.42%) | 17827(7.35%) |
| Medication history | Yes |  | 54767(22.58%) | 3601(1.48%) |

Table 3 : Association of Breast Cancer Risk with Allostatic load(N=181455)

|  | HR(95%CI) | Pvalue |
| --- | --- | --- |
| Age at recruitment | 1.05(1.03,1.07) | <0.01 |
| Race | 1.02(1.01,1.03) | <0.01 |
| white | Ref |  |
| black | 0.65(0.49,0.86) | <0.01 |
| asian | 0.87(0.71,1.07) | 0.18 |
| mixed or others | 0.70(0.55,0.90) | <0.01 |
| missing | 1.01(0.60,1.71) | 0.97 |
| Age when menarches | 0.99(0.97,1.00) | 0.13 |
| Age at first live birth |  |  |
| less than 30 | Ref |  |
| over 30 | 1.05(0.96,1.14) | 0.30 |
| missing | 1.12(1.05,1.19) | <0.01 |
| Ever taken oral contraceptive pill |  |  |
| yes | Ref |  |
| no | 1.01(0.94,1.08) | 0.80 |
| missing | 0.79(0.41,1.54) | 0.50 |
| Ever used hormone-replacement therapy (HRT) |  |  |
| yes | Ref |  |
| no | 1.09(1.03,1.16) | 0.01 |
| missing | 0.93(0.53,1.64) | 0.80 |
| Had menopause |  |  |
| yes | Ref |  |
| no | 0.90(0.81,0.99) | 0.03 |
| missing | 0.90(0.81,1.00) | 0.06 |
| Family History |  |  |
| no history | Ref |  |
| had history | 1.38(1.25,1.53) | <0.01 |
| missing | 1.03(0.97,1.09) | 0.29 |
| Education |  |  |
| high school or less | Ref |  |
| college / professional | 1.01(0.95,1.07) | 0.71 |
| missing | 0.90(0.83,0.97) | 0.01 |
| Employment Status |  |  |
| unemployment | Ref |  |
| employment | 0.99(0.89,1.09) | 0.79 |
| retired | 0.98(0.87,1.10) | 0.73 |
| missing | 0.87(0.63,1.20) | 0.39 |
| Income |  |  |
| Less than £30,999 | Ref |  |
| over ££30,999 | 1.08(1.01,1.15) | 0.03 |
| missing | 0.97(0.89,1.05) | 0.39 |
| Townsend Deprivation Score | 1.01(0.99,1.01) | 0.56 |
| Smoking |  |  |
| never | Ref |  |
| ever | 1.06(0.98,1.15) | 0.16 |
| missing | 0.89(0.53,1.47) | 0.64 |
| Total physical activity MET-hours/week |  |  |
| 0~20% | Ref |  |
| 20~40% | 1.01(0.92,1.11) | 0.82 |
| 40~60% | 1.05(0.96,1.16) | 0.26 |
| 60~80% | 0.88(0.80,0.97) | 0.01 |
| over80% | 0.88(0.79,0.97) | 0.01 |
| missing | 0.96(0.87,1.04) | 0.31 |
| Alcohol |  |  |
| special occasions or never | Ref |  |
| moderate | 1.02(0.95,1.10) | 0.62 |
| heavy | 1.15(1.07,1.24) | <0.01 |
| Sleeplessness |  |  |
| never/rarely | Ref |  |
| sometimes | 0.97(0.90,1.04) | 0.39 |
| usually | 0.98(0.90,1.06) | 0.56 |
| missing | 0.42(0.06,3.01) | 0.39 |
| Standard PRS for breast cancer | 1.72(1.67,1.76) | <0.01 |

Table 4 Association between individual biomarkers of AL scores and breast cancer risk.

|  | HR(95%CI) | Pvalue^1^ |
| --- | --- | --- |
| Higher Waist to hip ratio | 1.13(1.07,1.19) | <0.01 |
| Higher Pulse rate | 0.96(0.73,1.26) | 0.76 |
| Higher SBP | 1.08(1.02,1.15) | <0.01 |
| Higher DBP | 1.09(1.02,1.17) | 0.01 |
| Lower HDL | 1.13(1.06,1.21) | <0.01 |
| Higher Abnormal Cholesterol | 1.03(0.97,1.09) | 0.41 |
| Higher Triglycerides | 1.05(0.99,1.11) | 0.11 |
| Higher C-reactive protein | 1.13(1.06,1.20) | <0.01 |
| Higher Creatinine | 0.79(0.59,1.07) | 0.12 |
| Higher Glycated hemoglobin | 1.05(0.89,1.23) | 0.60 |
| History of metabolic disease or hypertension medication | 1.07(1.01,1.14) | 0.04 |

1: Adjusted by demographic variables, family history, reproductive factors, lifestyle factors, SES, and PRS
